# Evidence for a role of the ciliopathy protein MKS1 in cell polarity

**DOI:** 10.1186/2046-2530-4-S1-P42

**Published:** 2015-07-13

**Authors:** M Collado-Hilly, C Fisch, B Desforges, J Jerber, L Combettes, C Campillo, P Dupuis-Williams

**Affiliations:** 1 Genopole, ATIGE, Evry, France; 2 UMR 8587, CNRS, Evry, France; 3 UMR-S 757, INSERM, Orsay Cedex, France; 4 ESPCI, Paris, France

## 

Mutations in the protein MKS1 cause severe developmental disorders such as Meckel-Gruber syndrome (MKS). Dysfunctional MKS1 caused a panel of cellular defects ranging from abortive centriolar migration to ciliary instability and defective ciliary signaling [1-3]. Most analyses converge to the conclusion that in vertebrates, the depletion of MKS1 leads to impairment of Hh and Wnt signaling pathways [4-9]. Accordingly, MKS1 has been shown to be localized at the transition zone and as such, involved in the ciliary membrane composition [6].

By combining two complementary cell models -a mammalian epithelial cell line and the unicellular *Paramecium*, we identified a new function of MKS1 in cell polarity.

We show that MKS1 displays a typical pattern of membrane-associated protein, being localised to exocytotic vesicles, the plasma and the ciliary membrane and to cell junctions during epithelial differentiation. Based on RNAi experiments of MKS1 which leads to impairment of ciliary sensory functions, defective vesicle transport and plasma membrane distension, we propose that MKS1 knockdown impairs interactions between actin and membranes.

We finally show how MKS1 depletion interferes with epithelial differentiation and cell organogenesis in 3D cultures.
